# Supplementary material for: Field Use of Protective Bacteriophages against Pectinolytic Bacteria of Potato
Source: Microorganisms. 2023 Feb 28;11(3):620. doi: 10.3390/microorganisms11030620 (PMC10056506; doi:10.3390/microorganisms11030620)
Supplement: Supplementary file 1 [file microorganisms-11-00620-s001.zip › microorganisms-2240826-supplementary.pdf]

Supplementary Figure S1. Growth curves of bacteriophages φDs3CZ and φDs20CZ.

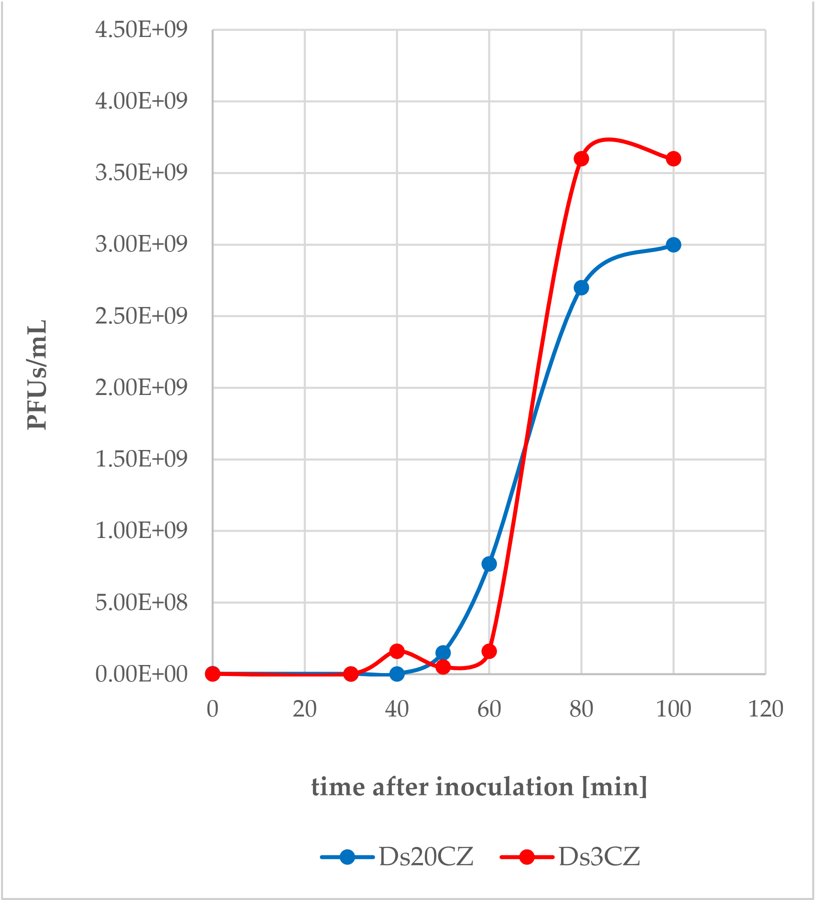

Supplementary Figure S2. Recombination analysis of Czech bacteriophage Ds25CZ (MN813053) genome. A) schematic representation of similarities between the Ds25CZ (MN813053) and the Coodle (MH807820) genomes. Blue boxes mark recombination events signalized by both RDP5 and alignment similarities; orange boxes mark highly different regions inferred from alignment only. B) Similarity values of individual parts of the Ds25CZ genome relative to all other limestoneviruses. The highest values indicating minor parents are boxed.

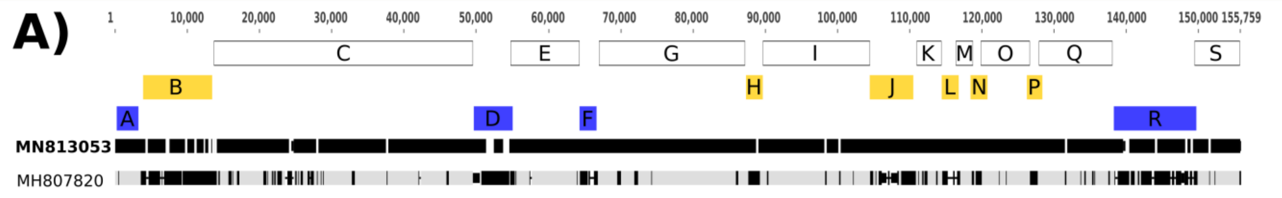

B)

	A	B	C	D	E	F	G	H	I	J	K	L	M	N	O	P	Q	R	S
KM209228 (justified)	82.04	97.82	97.20	60.63	99.98	74.90	80.57	**100.00**	99.14	99.42	99.79	99.91	**100.00**	**100.00**	98.45	**100.00**	84.00	91.71	98.45
KY942056	82.10	97.76	94.89	**62.84**	**99.99**	77.38	94.90	**100.00**	99.13	97.46	99.84	99.91	99.94	98.84	99.80	**100.00**	99.28	91.95	99.80
KY942057	82.21	91.67	92.83	**62.84**	99.98	75.55	95.10	**100.00**	99.04	72.81	99.79	56.59	99.94	90.90	99.80	79.98	**99.50**	77.71	99.80
MG266157	82.37	**97.88**	94.56	**62.84**	**99.99**	76.44	95.06	**100.00**	99.53	99.46	99.71	**100.00**	**100.00**	**100.00**	99.78	**100.00**	84.00	91.52	99.78
MH807812	82.18	92.99	96.21	62.38	99.98	**100.00**	92.21	99.93	99.03	99.47	99.79	99.87	**100.00**	**100.00**	99.78	**100.00**	84.01	**99.93**	99.78
**MH807820**	**99.95**	74.04	**97.79**	52.75	99.82	74.49	**99.78**	90.55	**99.75**	69.17	99.66	56.59	99.94	90.90	99.85	79.98	99.48	77.72	**99.85**
MN788369 (CR)	82.13	92.78	94.76	62.80	99.77	97.93	92.15	**100.00**	99.19	99.50	99.82	99.91	**100.00**	98.47	99.80	**100.00**	99.47	93.56	99.80
MN813048 (CR)	82.13	93.19	94.98	61.60	99.98	76.60	91.88	**100.00**	99.32	99.41	99.79	99.91	**100.00**	**100.00**	99.17	**100.00**	98.48	91.96	99.17
MN813049 (CR)	82.13	93.19	94.74	62.80	99.77	74.90	92.15	**100.00**	99.19	99.50	99.82	99.91	99.94	98.33	99.19	**100.00**	99.46	91.47	99.19
MN813050 (CR)	82.04	97.90	94.28	62.54	99.99	74.90	92.03	**100.00**	99.36	99.47	99.79	99.91	**100.00**	**100.00**	99.77	**100.00**	82.57	91.76	99.77
MN813051 (CR)	82.15	92.85	94.69	61.60	99.98	76.60	91.88	**100.00**	99.32	99.50	**99.90**	99.91	**100.00**	**100.00**	99.19	99.89	98.43	93.65	99.19
MN813052 (CR)	82.04	97.85	97.26	60.39	99.91	74.90	91.46	**100.00**	99.14	**99.54**	**99.90**	99.91	**100.00**	**100.00**	99.77	**100.00**	84.00	91.56	99.77
NC_019925	82.07	87.98	96.09	60.39	98.43	76.93	96.37	90.55	99.57	99.52	99.43	99.91	99.94	98.33	99.78	**100.00**	99.46	91.55	99.78
NC_025452 (justified)	82.02	77.29	96.00	61.01	98.44	76.08	96.30	90.55	92.39	**99.54**	99.69	56.59	99.94	98.33	99.80	79.98	84.00	91.50	99.80

Supplementary Figure S3. Curative effect of φDs3CZ and φDs20CZ solution in relation to concentration observed on tuber slices. Phage treatment at concentrations of 1 × 10^6, 1 × 10^7, and 1 × 10^8 PFUs/mL, negative control (water), and positive control (bacterial inoculation only). Positive control is essentially 100% macerated.

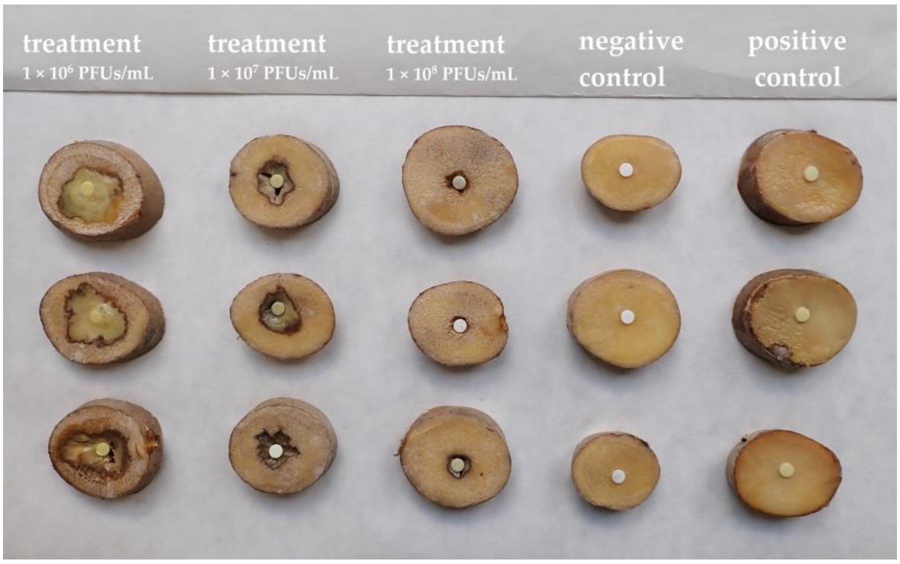

Supplementary Table S1. *Dickeya limestoneviruses* used for the recombination analysis

Isolate	AC number	nt	Country
Coodle	MH807820	152515	Denmark
Ds16CZ	MN813050	152837	Czech Republic
Ds20CZ	MN813051	154720	Czech Republic
Ds23CZ	MN813052	149364	Czech Republic
Ds25CZ	MN813053	151710	Czech Republic
Ds3CZ	MN788369	155285	Czech Republic
Ds5CZ	MN813048	154722	Czech Republic
Ds9CZ	MN813049	154716	Czech Republic
JA15	KY942056	153757	United Kingdom
Kamild	MH807812	152612	Denmark
Limestone	NC_019925	152427	Belgium
phiD3	KM209228	152308	Poland
PP35	MG266157	152048	Russia
RC-2014	NC_025452	155346	Poland
XF4	KY942057	151519	United Kingdom
